# The Clinical Relevance of Force Platform Measures in Multiple Sclerosis: A Review

**DOI:** 10.1155/2013/756564

**Published:** 2013-05-19

**Authors:** Luca Prosperini, Carlo Pozzilli

**Affiliations:** Multiple Sclerosis Centre, S. Andrea Hospital, Department of Neurology and Psychiatry, Sapienza University, Viale dell'Università, 30-00185 Rome, Italy

## Abstract

Balance impairment and falls are frequent in patients with multiple sclerosis (PwMS), and they may occur even at the earliest stage of the disease and in minimally impaired patients. The introduction of computer-based force platform measures (i.e., static and dynamic posturography) has provided an objective and sensitive tool to document both deficits and improvements in balance. By using more challenging test conditions, force platform measures can also reveal subtle balance disorders undetectable by common clinical scales. Furthermore, posturographic techniques may also allow to reliably identify PwMS who are at risk of accidental falls. Although force platform measures offer several theoretical advantages, only few studies extensively investigated their role in better managing PwMS. Standardised procedures, as well as clinical relevance of changes detected by static or dynamic posturography, are still lacking. In this review, we summarized studies which investigated balance deficit by means of force platform measures, focusing on their ability in detecting patients at high risk of falls and in estimating rehabilitation-induced changes, highlighting the pros and the cons with respect to clinical scales.

## 1. Introduction

Balance can be defined as the ability to maintain the body's centre of gravity (COG) within the base of support with minimal sway [1]. The control of human balance is a complex task which is assured by uninterrupted flow of afferent signals reaching the central nervous system (CNS) from the muscle, tendon and joint proprioceptors, skin exteroceptors, and vestibular and visual inputs [2]. The deficient integration of these pathways, due to the widespread and variable distribution of CNS damage in patients with multiple sclerosis (PwMS), can affect postural response and the ability to maintain adequate balance [3–5]. Balance impairment is frequently observed in PwMS, and it is among the most disabling symptoms [6]. A wide-base gait with worsening balance when changing direction has been often described in PwMS [7]. Fatigue, muscle weakness, and spasticity further contribute to compromise adequate balance and predispose them to accidental falls [8–11]. Fall tendency may occur early in the course of the disease, even before walking and balance impairment becomes clinically evident [12]. 

The incidence of accidental falls (i.e., an unexpected contact of any part of the body with the ground) in PwMS has been reported from 30% to 63% in a period variable from 1 to 12 months, according to different studies [13–27]. Recently, a large survey on about 195,000 veterans found a 2-fold increased risk of injurious falls in PwMS compared with sex/age-matched veterans without MS [28].

Studies investigating demographic and clinical characteristics related to a high risk of accidental falls in PwMS are quite heterogeneous in terms of sample size, setting, and design, and for reporting (retrospectively) or collecting (prospectively) the occurrence of falls [29]. Studies relying on retrospectively collected patient report of falls at the inclusion are prone to recall bias [16, 30], although a good correlation (*r* = 0.82) between prospectively and retrospectively collected falls has been demonstrated [16]. In addition, even if prospectively collected, falls resulting in injury are more likely to be reported, and cognitive or memory impairment may further decrease the accuracy of their recall [16, 18]. 

From a clinical point of view, reliably discriminating fallers between nonfallers is crucial for the development of a program aimed at fall prevention. Potentially, force platform measures may provide an objective, reliable, and accurate tool for this purpose. Moreover, they may be useful for documenting not only deficits but also improvements of balance skills after specific intervention.

In this review, we aimed to summarize studies investigating the role of force platform measures in MS setting, focusing on (i) differences between PwMS and general population; (ii) ability in detecting PwMS at high risk of falls, highlighting also the differences with clinical scales; (iii) evaluation of rehabilitation-induced changes.

### 1.1. Clinical Scales to Assess Balance

Clinical tests usually rate balance performance on a set of motor tasks. Scoring is based on the sum of ordinal item scores or stopwatch measurements. Ideally, an evaluation of postural balance should include clinical scales that are practical, sensitive selective, reliable, and valid. Although some clinical scales are easy and relatively quick to use, they are hampered by their variable execution and by the room left for evaluator judgment in the scoring system [31, 32].  Table 1 summarizes the most commonly used clinical scales to assess balance in PwMS and their main psychometric properties [33–41].

So far, few studies provided data on diagnostic accuracy of clinical scales in detecting PwMS prone to accidental falls. These studies showed conflicting results, probably due to different cutoffs established (see also Table 1). Cattaneo and colleagues [14] showed that clinical balance scales exhibit good specificity (i.e., performance in detecting nonfallers), but low sensitivity (i.e., performance in detecting fallers). Although other authors found differences between fallers and nonfallers in clinical scale scores of balance and even mobility [20, 22, 23, 25], they did not provide data on sensitivity and specificity. Nilsagård and colleagues [16] suggested a combination of patient variables and selected clinical scales to predict the risk of falls but failed to identify the “best candidate” to apply in the daily setting. More recently, it has been suggested that the BESTest was 92% accurate in identifying fallers and nonfallers among PwMS [35]. Despite this high accuracy, the BESTest is time consuming and requires a lot of tools. The use of a short version (mini-BESTest), having only a 10-minute administration time, could be more useful in clinical practice, but it needs to be validated in PwMS [42]. Lastly, an association between cognitive processing speed and fall frequency has been recently described in PwMS [43]. D'Orio and colleagues [23] also suggested that cognitive impairment, especially impaired verbal memory, predicted an increased risk of recurrent falls.

### 1.2. Force Platform Measures: Basic Principles

Force platforms are instruments that measure ground reaction forces generated by a body standing on or moving across them, to quantify biomechanical parameters of human balance control. Force platforms are also used for gait analysis.

Posturography is the general term encompassing all the techniques used to quantify postural control in upright stance, in either static or dynamic conditions, by means of a force platform [44]. The term static posturography refers to the characterization of postural sway of the centre of pressure (COP) (i.e., the point of application of the resultant from the vertical force's action) during quiet standing on a fixed support surface (i.e., a relatively unperturbed state). In quiet stance, the COP is estimated as compatible with the centre of gravity at about 97%; this compatibility diminishes in dynamic condition [45]. Variations in the instant positions of the COP during a 30- or 60-second test are used to calculate time-domain measures, including the velocity of the COP on the anteroposterior or mediolateral axes (mm/s), the sum of the displacements (path) of COP (mm), and the 95% confidence ellipse area of COP (mm^2^). From abiomechanical standpoint, the displacement of the COP represents a marker of energy expenditure to maintain balance [46]. Usually, a posturographic assessment consists in two test conditions (eyes opened and closed) and, sometimes, in dual-task condition [47]. This paradigm allows an evaluation of cognitive processing required to maintain standing balance, simply by applying a concurrent cognitive task (e.g., aloud or silent backward counting, Stroop test, and paced auditory serial addition test). 

Static posturography provides linear, objective, and reliable measurements of static balance [44]. In spite of its reliability and accuracy in PwMS [24, 48], the main limitation of static posturography is a lack of standardisation that precludes the possibility to generalize its application for multicentre purposes. This is due to the fact that different force platform equipment and different test procedures are used in clinical practice. Parameters that should be considered are not well defined (e.g., velocity, path, area, etc.), as well as feet position and test duration [49]. Additionally, static posturography evaluates balance control only in the most simplistic condition, thus not reflecting situations occurring in daily-life activities.

Dynamic posturography involves the use of experimentally induced (external or self-generated) balance perturbation, such as shifting the support surface, using an unstable support surface, moving the visual surround, applying stimuli to upper body parts, and performing voluntary weight shift [50]. By manipulating one or more specific inputs (visual, vestibular, or proprioceptive) for postural control, a dynamic posturography assessment may provide important data on the motor and sensory contribution to balance control [51]. Thereby, impairments in sensory reweighing and integrating afferent inputs can be easily detected. Moreover, these data can be combined into composite scores, such as the equilibrium score or the postural stability index [52]. The main advantage of dynamic posturography is the possibility to obtain information on balance control in a variety of conditions simulating situations encountered in daily-life activities [32]. Unfortunately, it requires a long time of administration and an expensive and bulky equipment. Moreover, subjects cannot maintain balance under the more difficult conditions, especially when they are forced to rely only on vestibular input. A fall frequency as high as 22% has been reported while PwMS performed the more challenging conditions of dynamic posturography (i.e., surface moving, eyes opened; surface moving, eyes closed; surface and surround moving, eyes opened) [48].

## 2. Methods

### 2.1. Data Sources

PubMed was searched for abstracts using the following medical subject heading (MeSH) terms: “multiple sclerosis” AND “posturography” OR “multiple sclerosis” AND “force platform” OR “multiple sclerosis” AND “postural balance.” No limitations or time period restrictions were applied and the latest search was undertaken on January 10th 2013, Both prospective and retrospective studies were encompassed. Published conference abstracts,case reports, meta-analyses and reviews, articles not available in English, and studies including also patients affected by neurological conditions other than MS were excluded. Finally, studies where postural sway was measured by means of tools (e.g., accelerometers or gyroscopes) other than force platforms were also excluded. Abstracts of resulting articles were then hand searched in order to select studies which met eligibility criteria. Attempts to identify further articles were done by searching for the references of the studies. 

## 3. Results

The search initially yielded a total of 178 articles; out of these, 58 studies conducted on PwMS were selected for this narrative review. After removing duplicates, 35 met the inclusion criteria. In 21 studies, force platform measures were used to detect impairments in balance in PwMS with healthy subjects as control group [48, 53–72]. Five studies investigated the role of force platform measures in detecting fall status of PwMS [17, 19–21, 24]. Finally, 9 studies used force platform measures as outcome measures to determine the effectiveness of a rehabilitative intervention [73–81].

### 3.1. Differentiating Balance Control between Patients with MS and Healthy Subjects

There is a general agreement that PwMS have a postural sway control which is significantly poorer than healthy subjects. PwMS present larger oscillations in the frontal and sagittal planes when compared with healthy controls [17, 19–21, 24, 48, 53–71]. By means of posturography, impaired anticipatory postural adjustments have been also described in PwMS [69].

Furthermore, the sensitivity of force platform measures is such that it can detect balance abnormalities even in minimally impaired PwMS (i.e., scoring as normal in clinical balance test) [57, 63, 68] or in those presenting a first demyelinating event suggestive of MS [64]. This latter study demonstrated that about 40% of CIS patients had poor or very poor scores in COP sway rate (i.e., 2–4 or ≥4 standard deviations higher than the mean value of healthy controls, resp.) [64]. Therefore, posturography demonstrates the existence of subclinical balance disorders that cannot be detected by means of clinical assessment, even in PwMS who did not complain about subjective balance impairment [68].

Another common finding of these studies is that postural stability deficit is increased under more challenging conditions, for example, reducing the base of support, suppressing visual or vestibular input, generating external perturbations, and performing a reach and lean task or a cognitive task [17, 54, 59, 62, 64, 68]. It has also been shown that an abnormal performance in quiet standing can be found in 2/3 of PwMS, even when all sensory inputs (visual, vestibular, and proprioceptive) are available; the alteration of a single input can lead to an increase in abnormal findings by up to 82% [48]. 

### 3.2. Predicting the Risk of Future Falls

**Figure 1:**
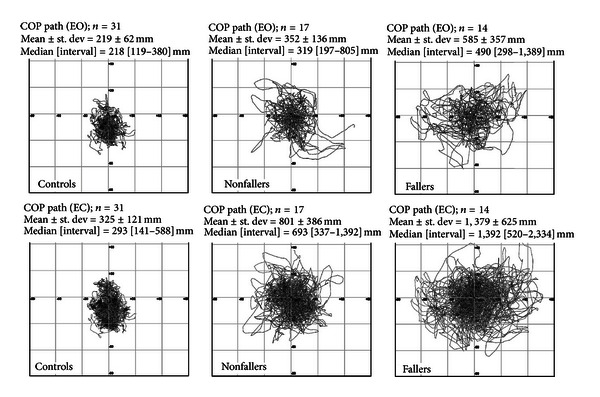
Superimposed displacements of centre of pressure (COP path) on *x*-*y*-axes with both eyes opened (EO) and closed (EC) (upper and lower rows, resp.) of healthy volunteers (controls, *n* = 31), patients without a history of falls (nonfallers, *n* = 17), and those reporting one or more falls in the past 6 months (fallers, *n* = 14) (modified from [19]).

Up to now, only a few studies investigated the role of force platform measures in predicting the risk of falls in PwMS. Several studies reported fallers as having wider COP sway than nonfallers [19, 20, 24]. Therefore, a gradient of postural disturbance can be hypothesized as follows: PwMS fallers > PwMS nonfallers > healthy subjects (Figure 1) [19, 24]. However, this hypothesis needs to further confirmations.

Sosnoff and colleagues [20] showed that PwMS classified as fallers exhibited increased COP sway velocity in the mediolateral direction under eyes opened condition, wider overall COP sway area, and greater sway velocity in the anteroposterior and medio-lateral directions under eyes closed condition. Other studies provided similar findings, with fallers' COP moving more and faster in either anteroposterior or mediolateral directions than nonfallers, in both eyes opened and closed eye conditions [19, 24].

Kasser and colleagues [17] demonstrated that women with MS who experienced accidental falls were correctly identified by dynamic posturography, which was able to discriminate patients reporting at least one fall over the past 12 months from those reporting more frequent falls. Impaired forward limit of stability, gait asymmetries, and leg flexor-extensor muscle weakness also contributed to detecting recurrent fallers.

**Figure 2:**
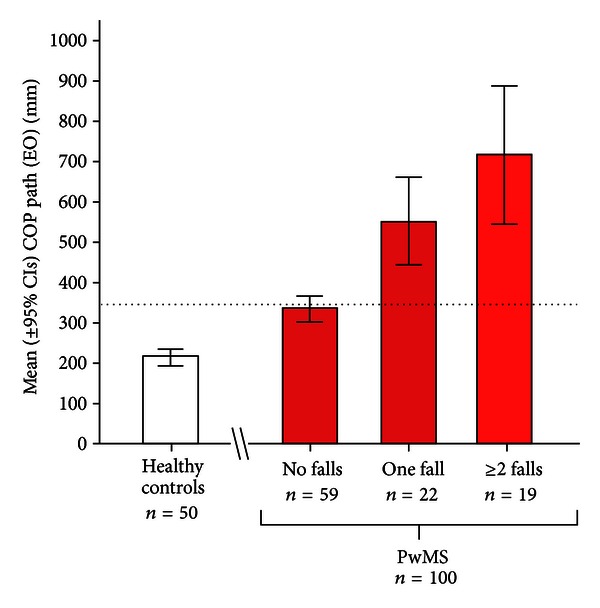
Mean (±95% confidence intervals) values of centre of pressure (COP) path with eyes opened (EO) of healthy volunteers (*n* = 50) and patients with MS (*n* = 100) who were divided according to the number of accidental falls (0, 1, ≥2) prospectively collected over a 3-month follow-up period (modified from [24]).

Only one recent study supports the notion that the adjunction of posturographic evaluation did not improve the ability to detect PwMS prone to fall [21]. However, as also recognized by authors, there are some limitations to their study: (i) a small sample size (*n* = 37); (ii) the incidence of falls was lower compared to other published papers, probably due to the short observational time-frame considered (2 months); (iii) they did not use traditional force platform measures as outcome, but the derivative virtual time to contact (i.e., the time taken by COP to reach the stability boundaries). Recently, we estimated sensitivity, specificity, and accuracy of static standing balance measures in predicting patients who experienced future falls [24]. We examined 100 consecutive PwMS by means of neurological examination (including the Berg scale) and a static posturography assessment. The patients were instructed to report the occurrence of falls over the next 3 months. Balance measures above the mean plus 2 standard deviations of normal values (as provided by a sample of 50 sex-/age-matched healthy volunteers) were considered as abnormal. From static posturography, the COP path under open eye condition was extrapolated, providing a measure not only highly reliable (95%) but also more sensitive (88% versus 37%) and accurate (75% versus 63%), but slightly less specific (67% versus 81%) than a common clinical test (such as the Berg scale) in predicting accidental falls. A multivariate logistic regression analysis revealed that there was an 8%-increased risk of being classified as fallers for each 10-mm increase of COP path value, even after adjusting for other demographic and clinical variables. Finally, a “dose effect” of static posturography was also found; that is the wider the COP path, the greater the number of accidental falls prospectively recorded by PwMS (Figure 2) [24].

### 3.3. Evaluating Rehabilitation-Induced Changes

Force platform measures ensure an objective, reliable, and linear assessment of balance, avoiding the risk of ceiling effect [32]. Force platform measures demonstrated a high sensitivity in detecting rehabilitation-induced changes and sometimes provided more rewarding results than clinical scales [74–77, 79–81]. Concurrent improvements in postural sway measures, clinical, and/or patient-reported outcomes were always described [74–77, 79–81]. Only two studies did not show any improvement of postural sway measures of PwMS after home-based resistance exercises [73] and balance-based torso weighting [78].

One recent study aimed at investigating the effectiveness of a 12-week home-based balance training using a commercial videogame platform showed also a slight increase in the proportion of nonfallers when compared with the 3-month period prior to study enrolment [81]. However, this latter study was not designed (and not powered to perform a post hoc analysis) to estimate a relationship between force platform measure changes and clinically relevant outcomes.

Unfortunately, there are still no data on the clinimetric property [82] of responsiveness of force platform measures, assessed by minimally important change (MIC) over time (i.e., the change that is relevant for the patient) and smallest real change (SRC) (i.e., the change on a measurement instrument required to overcome the measurement error). Therefore, future research efforts are warranted to establish MIC and SRC for force platform measures.

## 4. Conclusions

Balance impairment and falls are frequent in PwMS, and they may occur even at the earliest stage of the disease. Reliably identifying subjects who are at risk of accidental falls is a clinical challenge. Asking about the presence of prior falls is unreliable because patients often neglect their falls. Clinical balance scales are hampered by their variable execution and subjective scoring system, thus providing conflicting results about their ability to detect patients prone to falls.

By contrast, within few minutes, computer-based force platform measures of standing and dynamic balance can provide useful information regarding the risk of future falls, as well as intervention-induced changes. Moreover, computerised postural sway measures have been reported as correlated with disability and functional scales [24, 35, 65, 83].  Table 2 summarizes pros and cons pros of force platform measures, contrasted with those of clinical measures.

Although relevant, the differences in postural control between PwMS and healthy subjects cannot definitively elucidate the neuropathological mechanisms leading to balance impairments in MS. Given the widespread and variable distribution of CNS damage, it is generally thought that postural control impairment in PwMS has multifactorial causes that differ from one person to the next [4, 17]. Studies investigating the structure-function relationship by means of force platform measures do not provide comparable results. Jackson and colleagues [55] suggested that postural balance deficit in PwMS resulted from impaired central integration of visual, vestibular, and somatosensory input. Slowed afferent proprioceptive conduction along demyelinated dorsal columns of spinal cord has been proposed as an important cause of impaired postural control [84, 85]. Another hypothesis proposes the damage of cerebellar connections (i.e., cerebellar peduncles) as the primary contributor to the balance impairment [19] or, more extensively, the focal and diffuse involvement of the cerebellum, its connections, and other associative regions [5]. 

### 4.1. Future Recommendations

Posturographic systems have become more affordable and potentially useful for both clinical practice and research purposes. Nevertheless, they still represent a significant cost (especially dynamic posturography equipment) need a dedicated space and trained staff to run the tests. This is not always feasible in a clinical practice setting. A possible solution to overcome the main drawbacks of laboratory-grade force platforms could be the implementation of software to interface a commercial Nintendo balance board with a common personal computer [86]. Similarly to laboratory-grade force platform, the balance board contains force sensors which detect subject's COP and weight shifts. This commercial device—that has been recently included in the neurorehabilitation process of PwMS [79–81]—is low expensive, portable, and user friendly. In conclusions, further efforts are warranted to establish (i) which parameters of balance (velocity, path, area, etc.) should be evaluated; (ii) normative values for the force platform measures; (iii) how to standardize the posturographic assessment for multicentre study purposes; (iv) the ecological validity of this tool.

## Figures and Tables

**Table 1:** Commonly used clinical scales to assess balance in patients with multiple sclerosis.

Tool	Authors Journal	Brief description	Time of administration	Overall score	Test-retest reliability in PwMS	Accuracy in predicting fall status in PwMS
Activities-specific balance confidence (ABC)	Powell and Myers [33] J. Gerontol. A. Biol. Sci. Med. Sci. 1995	16-item self-administered questionnaire rating the perceived level of confidence in performing daily living activities	15 minutes	0 to 100	92%	SE: 65%, SP: 77% (cutoff: 40) [14]
Balance evaluation system test (BESTest)	Horak et al. [34] Phys. Ther. 2009	36-item physician-rated scale evaluating 6 systems (biomechanical constraints, stability limits/verticality, anticipatory postural adjustments, postural responses, sensory orientation, and stability in gait)	30 minutes	0 to 108	88%–91%^b^	SE: 86%, SP: 95% [35]
Berg balance scale (BBS)	Berg et al. [36] Can. J. Public Health. 1992	14-item physician-rated scale exploring the ability to sit, stand, lean, and turn and postural transition.	15 minutes	0 to 56	96%	SE: 40%, SP: 90% (cutoff: 44) [14] SE: 94%, SP: 32% (cutoff: 55) [16] SE: 32%, SP: 87% (cutoff: 44) [24]
Dizziness handicap inventory (DHI)	Jacobson and Newman [37] Arch. Otolaryngol Head Neck Surg 1990	Multidimensional 25-item self-administered questionnaire quantifying the level of disability in three domains: physical, emotional, and functional	15 minutes	0 to 100^a^	90%	SE: 50%, SP: 74% (cutoff: 59) [14]
Dynamic gait index (DGI)	Whitney et al. [38] J. Vest. Res. 2000	8-item physician-rated scale exploring mobility function and dynamic balance	10 minutes	0 to 24	85%	SE: 45%, SP: 80% (cutoff: 12) [14]
Four-square step test (FSST)	Dite and Temple [39] Arch. Phys. Med. Rehabil 2002	Stop-watch measurement of the duration of rapidly step over low obstacles in clockwise and counterclockwise direction	3 minutes or less	N/A	93%–98%^b^	SE: 60%, SP: 75% (cutoff: 16.9 s) [16]
Functional reach test (FRT)	Duncan et al. [40] J. Gerontol. 1990	Measurement of the maximum distance reached forward while standing in a fixed position.	N/A	N/A	85%–95%^b^	—
Timed-up-and-go test (TUG)	Podsiadlo and Richardson [41] J. Am. Geriatr. Soc. 1991	Stop-watch measurement of the duration of standup from a chair, walking 3 meters, turning around, walking back and siting down.	3 minutes or less	N/A	98%	SE: 73%, SP: 54% (cutoff: 13.6 s)^c^ [16]

PwMS: patients with multiple sclerosis; SE: sensitivity; SP: specificity; ^a^the only scale in which the lower the score, lower the level of disability; ^b^as estimated in populations other than MS; ^c^cognitive TUG was used in this study.

**Table 2:** Summary of pros and cons of force platform measures and clinical scales.

	Force platform measures	Clinical scales
Equipment		
Expensive	Y	N
Cumbersome	Y	N
Training of staff required	Y	Y/N^a^
Data collection		
Easy and fast to administer	Y/N^b^	Y/N^b^
Affected by emotional status or external factors	Y	Y
Invasive for patients	N	Y/N^c^
Statistical consideration		
Linear values	Y	N
Objective measurements	Y	N
Ceiling effect	N	Y
Reliability	Y	Y
Clinical utility		
Detection of subclinical impairment	Y	N
Identification of underlying causes of imbalance	Y/N^d^	Y/N^d^
Prediction of falls	Y	Y
Ability in detecting improvements	Y	Y

^a^Self-administered questionnaire did not require any specific training; ^b^BESTest and dynamic posturography may be time consuming; ^c^dynamic posturography may be poor tolerated; ^d^BESTest and dynamic posturography can identify the system that mainly affect balance.
